# Improving visualization and quantitative assessment of choriocapillaris with swept source OCTA through registration and averaging applicable to clinical systems

**DOI:** 10.1038/s41598-018-34826-5

**Published:** 2018-11-14

**Authors:** Zhongdi Chu, Hao Zhou, Yuxuan Cheng, Qinqin Zhang, Ruikang K. Wang

**Affiliations:** 1 Department of Bioengineering, University of Washington, Seattle, Washington USA; 2 Department of Ophthalmology, University of Washington, Seattle, Washington USA

## Abstract

Choriocapillaris (CC) visualization and quantification remains challenging. We propose an innovative three-step registration and averaging approach using repeated swept source optical coherence tomography angiography (SS-OCTA) scans to conduct automatic quantitative assessment on CC. Six subjects were enrolled, each imaged at several locations with SS-OCTA from macular to equatorial regions using 3 mm × 3 mm scanning pattern. Five repeated volumes were collected for each subject. The complex optical microangiography (OMAG) algorithm was applied to identify blood flow in CC slab. An automatic three-step registration of translation, affine and B-Spline was applied to *en face* OCTA images of CC, followed with averaging. A fuzzy clustering approach was used to segment vasculature and flow deficits from the averaged images. The improvement in visualization of CC was evaluated and the average intercapillary distance was estimated by calculating the averaged capillary lumen spacing. A series of quantitative indices of flow deficit density, number, size, complexity index and aspect ratio index (FDD, FDN, FDS, FDCI and FDARI) were designed and validated with the increase of repeated scan numbers for averaging. Quantitative assessment was applied and compared on CC in macular and equatorial regions. The intercapillary distance was observed to be around 24 µm at macula and increased toward equatorial regions. All five quantitative indices (FDD, FDN, FDS, FDCI and FDARI) showed significant changes with multiple averaging and tend to become stable with repeated number of 4. Our proposed registration and averaging algorithm significantly improved the visualization of CC with SS-OCTA. The designed five indices for CC provide more options in the quantitative assessment of CC and are of great potentials in assisting the understanding of disease pathology, early diagnosis and treatment monitoring.

## Introduction

Choriocapillaris (CC) is a thin physiological layer permeated with dense capillary networks, located at the inner choroid between Bruch’s membrane and Sattler’s layer. Previous studies^1–4^ have reported close correlation between abnormal CC circulation and multiple retinal/choroidal diseases such as age-related macular degeneration (AMD), diabetic retinopathy (DR), glaucoma etc. Therefore, improving the visualization of CC and its quantitative assessment is of great importance for advancing our understanding of diseases’ pathology, early diagnosis and treatment monitoring.

The microvascular network in CC has distinguishing morphologies at different regions. Histological studies^5–8^ have shown that CC appears as a dense honeycomb pattern of freely interconnected capillaries separated by septa within the submacular region, while in posterior pole, equatorial and peripheral regions, CC shows a lobular pattern where feeding arterioles and draining venules from deeper choroid join the segment from either the center or on the periphery of lobule^8^. The CC vessel diameter was measured to be 16–20 µm under the macular^5,8^. Dye-based angiography such as fluorescein angiography and indocyanine green angiography have been used to evaluate CC and observed similar patterns to histological images, but their limited resolution and effects of dye leakage prevent us from visualizing the CC at microscopic level^9^.

As a non-invasive imaging technology that uses repeated B-scans to contrast blood flow motions in tissue, optical coherence tomography (OCT) based angiography (OCTA)^10–12^ has drawn increasing attention recently for CC imaging^13–25^. Kurokawa *et al*.^26^ reported successful CC imaging using adaptive optics OCT (AO-OCT) with a 2.4 µm lateral resolution. Their home-built system achieved 0.75 and 1.5 μm/A-scan sampling rate along slow and fast scan direction respectively. Moreover, Gorczynska *et al*.^13^ also demonstrated CC imaging using a 1.7 MHz swept source OCT (SS-OCT) with a 14 µm lateral resolution. This high-speed system enabled high lateral sampling rate (4 µm/pixel) and large number of repeated B-scans (10 repeats). Such designs successfully suppressed the noise caused by multiple scattering due to retinal pigmented epithelium (RPE) in CC imaging. However, most commercially available OCTA systems have lower lateral resolution (15–20 µm), larger pixel sampling rate (> = 10 µm/pixel) and fewer repeated B-scans (2–4 repeats). Such system specifications have demonstrated satisfactory abilities for retinal vasculature imaging, but fall short of reliable visualization and quantification of the CC.

Within the confinement of commercial OCTA systems, post-processing technologies could be helpful to improve the quality of CC imaging. Recently, several studies^21,27,28^ have demonstrated that registering and averaging multiple *en face* OCTA images could significantly improve image quality in both retina and CC with SD-OCT, yet the reported method requires a large number of repeated scans and relies on the integrity of normal retinal vasculature. Due to the complexity of CC vasculature, flow deficit (FD) area density has been used as an index to quantify CC with SS-OCT and was found to be closely correlated to diseases^19,21,29,30^. Zhang *et al*. used a normal database to assist the quantification of CC FD, while this approach is plausible, collecting such comprehensive database among multi-centers globally remains time consuming and challenging. Al-Sheikh *et al*. used Phansalkar’s local thresholding to segment CC vasculature from flow deficits, yet their segmented image seems to underestimate vasculature and overestimate FDs. Moreover, more indices for quantitative assessment of CC are in demand for clinical studies and evaluation of treatments.

In this study, we propose a novel approach to compensate the motion pattern of OCT systems with a three-step registration including translational registration, affine registration and B-spline registration, followed with averaging. This will largely improve the image quality of CC for better visualization. For CC quantitative assessment, we also propose several reliable indices based on the resulting *en face* OCTA images of the CC. These indices include FD density, number, size, complexity index and aspect ratio index, and also with which to generate additional functional maps of FD binary map, perimeter map, length map, aspect ratio map, complexity map and size map. We hope these assessments will be of great help to quantitative analysis on the CC vasculature and its correlation with various diseases.

## Methods

This cross-sectional, observational case series was approved by the Institutional Review Board at the University of Washington and was conducted following the tenets of the Declaration of Helsinki and the Health Insurance Portability and Accountability Act of 1996 regulations. Informed consents were obtained from all subjects before participation.

### SS-OCTA imaging and CC slab generation

SS-OCT data were obtained using a PLEX® Elite 9000 (Carl Zeiss Meditec, Dublin, California) with a 1060 nm central wavelength and a bandwidth of 100 nm. This system provides an axial resolution of ~5.5 µm in retinal tissue and a lateral resolution of ∼20 μm estimated at the retinal surface^31^. 3 mm × 3 mm scanning protocol was used for imaging, with 300 A-lines per B-scan and 300 B-scans repeated 4 time each location per volume. This provides a 10 µm/pixel sampling rate transversally. Recruited subjects were scanned at the fovea and in the equatorial region just outside of infero-temporal arcade. Complex optical microangiography (OMAG^c^) algorithm^32^ was used to generate OCTA volume and a semi-automated segmentation algorithm^33^ was used to perform layer segmentation with necessary manual correction when needed. CC slab was defined as a 15 μm thick slab, starting 16 μm under the RPE^13,15^. *En face* CC OCTA images were generated using sum projection and linear display^15^. Standard image pre-processing was applied such as local illumination normalization^34^ to compensate for uneven illumination caused by overlaying anatomy such as RPE, floaters or vitreous opacity.

### Registration and averaging

Due to inevitable subject movement, repeated volume scans are required to be registered before averaging. Intensity based registration can be described as a convex optimization problem:

1 $$\begin{array}{c}\,\hat{T}=argmin\,C({T}_{\mu };\,{I}_{F},\,{I}_{M})\end{array}$$

where *I*_*F*_ is the reference (fixed) image and *I*_*M*_ is the moving image to be registered. *T*_*μ*_ represents the registration transformation function with parameters *μ*. *C* is the mutual information cost function that quantifies the joint entropy of variables, and measures the difference between the fixed and registered images. The goal of optimization is to find the optimal transformation coordinate parameters *μ*, that can minimize the difference between the reference image and moving image.

To compensate the motion pattern of OCT scans, we adopted a three-step registration approach that includes translational registration, affine registration and B-spline registration. This software is completely automatic and developed in house (R2016b; MathWorks, Inc, Natick, Massachusetts, USA). Translational registration roughly eliminates large horizontal and vertical misplacement of two OCTA CC images. Then, affine transformation was performed to compensate for geometric distortions such as scaling rotation and shearing between two CC images. Lastly, non-rigid B-spline registration was performed to further refine the registration within the sub sectional images.

**Figure 1:**
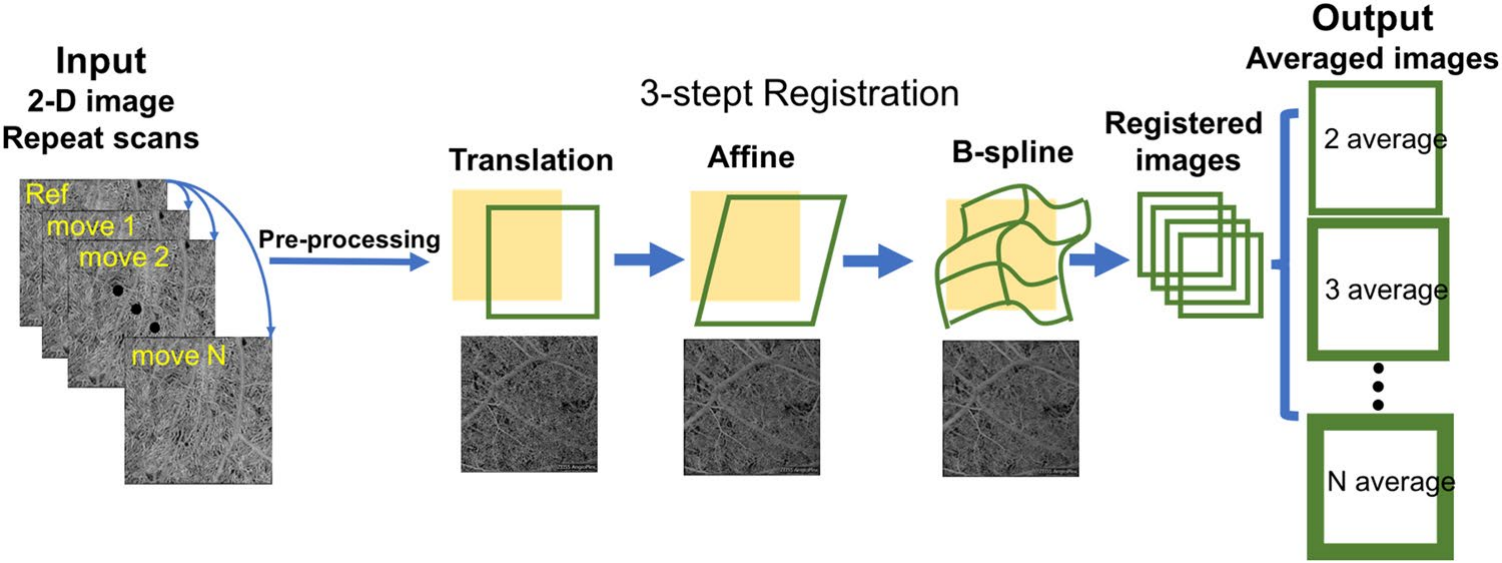
Illustration of the registration and averaging method to improve the image quality of the choriocapillaris. A series of repeated projection images were successively performed translation, affine and B-spline registration with the reference to the first scan, to eliminate the deformation and motion between images.

Once the images were registered, averaging can be performed to improve CC image quality for further quantification. For each subject, we acquired single scan image, 2-scan averaged image, 3-scan averaged image, 4-scan averaged image and 5-scan averaged image. The computational process is described in Fig. 1. Parameters of global entropy^35^, global standard deviation, local texture correlation^36^ and peak signal to noise ratio (PSNR)^37^ were used to evaluate the improvement of registration and averaging algorithm.

### Intercapillary distance measurement

Power spectrum analysis has been widely used to determine collagen fiber orientation^38^, this method has also been adopted to assess average CC lumen spacing with both AO-OCTA and SS-OCTA^13,26^. Here, we used it to measure intercapillary distance (ICD) in macular CC and equatorial CC. Multiple areas of 650 µm × 650 µm region with clear CC vasculature and no motion artifacts were selected from three subjects in macular, posterior pole and equatorial regions. A two-dimensional power spectrum was generated and radially averaged. The cusp in the radially averaged plot represents the most prevalent spacing, which is the averaged intercapillary distance in the CC image.

### Fuzzy c-means clustering for CC segmentation

**Figure 2:**
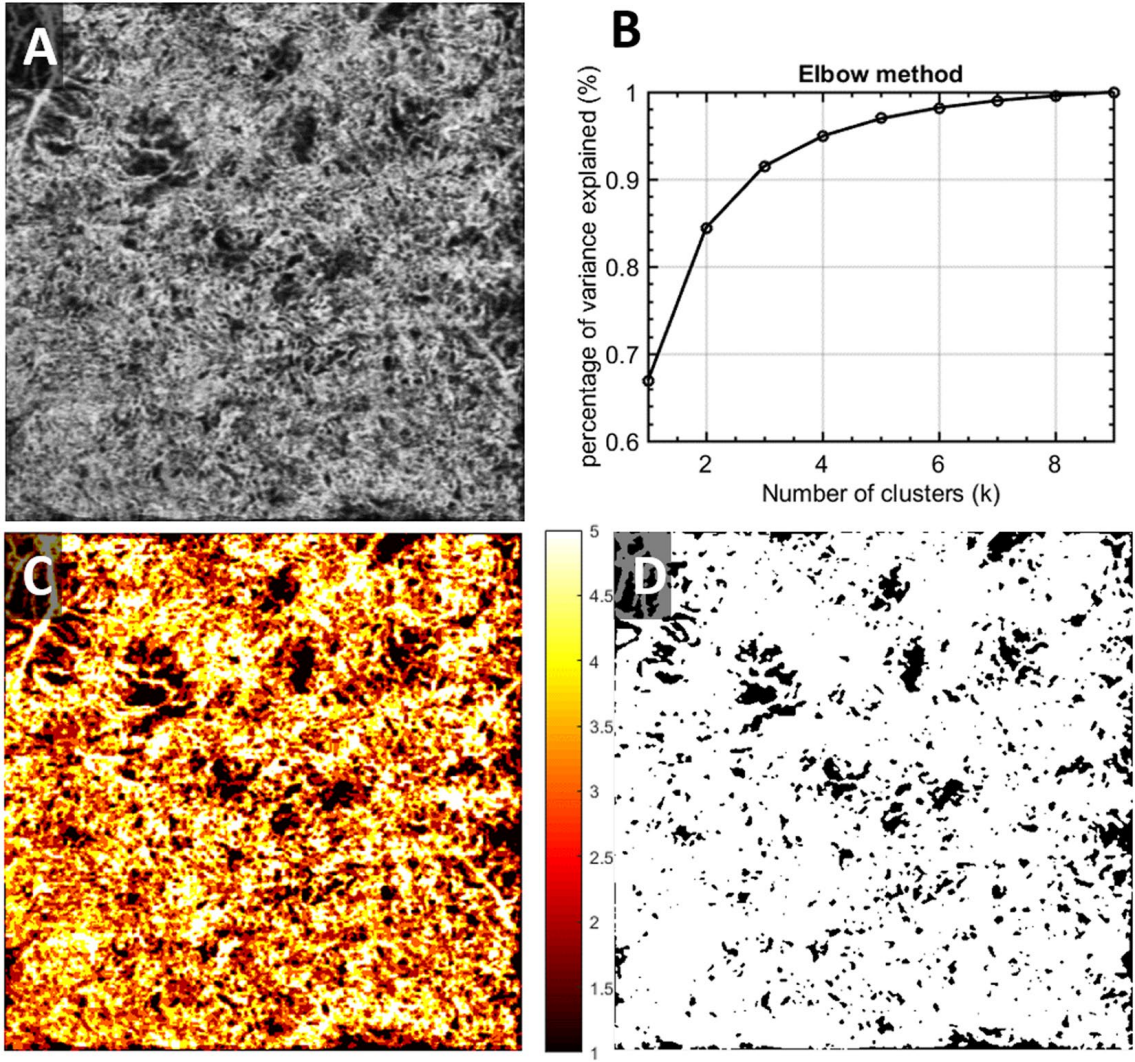
Illustration of the CC FDs segmentation using fuzzy c-means on a chronic birdshot chorioretinopathy patient. (**A**) Averaged OCTA CC image; (**B**) elbow method to determine appropriate number of clusters; membership is designated to each cluster of signals; (**C**) fuzzy c-means membership map of original CC image, all pixels were assigned into 5 different memberships based on fuzzy logic; (**D**) binary CC FD map, pixels presented in black represent identified CC FDs.

After registration and averaging, OCTA *en face* images of CC (Fig. 2A) were used for further quantitative assessment. Note that for better illustration purposes, we used scans from a patient diagnosed with chronic birdshot chorioretinopathy. We adopted a fuzzy clustering approach, Fuzzy c-means (FCM) to segment vasculature and FDs in CC, for its widely tested and validated efficiency in medical image segmentation, flexibility in membership determination, and practical spatial constraints^39,40^. This approach adjusts initial clusters based on partial membership of each site to other clusters to form final clusters that meet the criteria of homogeneity. Elbow method was applied to determine the optimal number of memberships for clusters^41^. That is, to increase the number of clusters until the variance between clusters explained over 99% of total variances (Fig. 2B). In the resulting membership map (Fig. 2C), each pixel’s value was optimized from their original intensity to represent their membership (1–5 in this case) and was color-coded according to their membership number. CC FD map is generated by segmenting the first membership (Fig. 2D). After removing the projection artifacts^42^, this binary CC FD map is used for further quantifications.

### Quantitative indices for CC assessment

**Figure 3:**
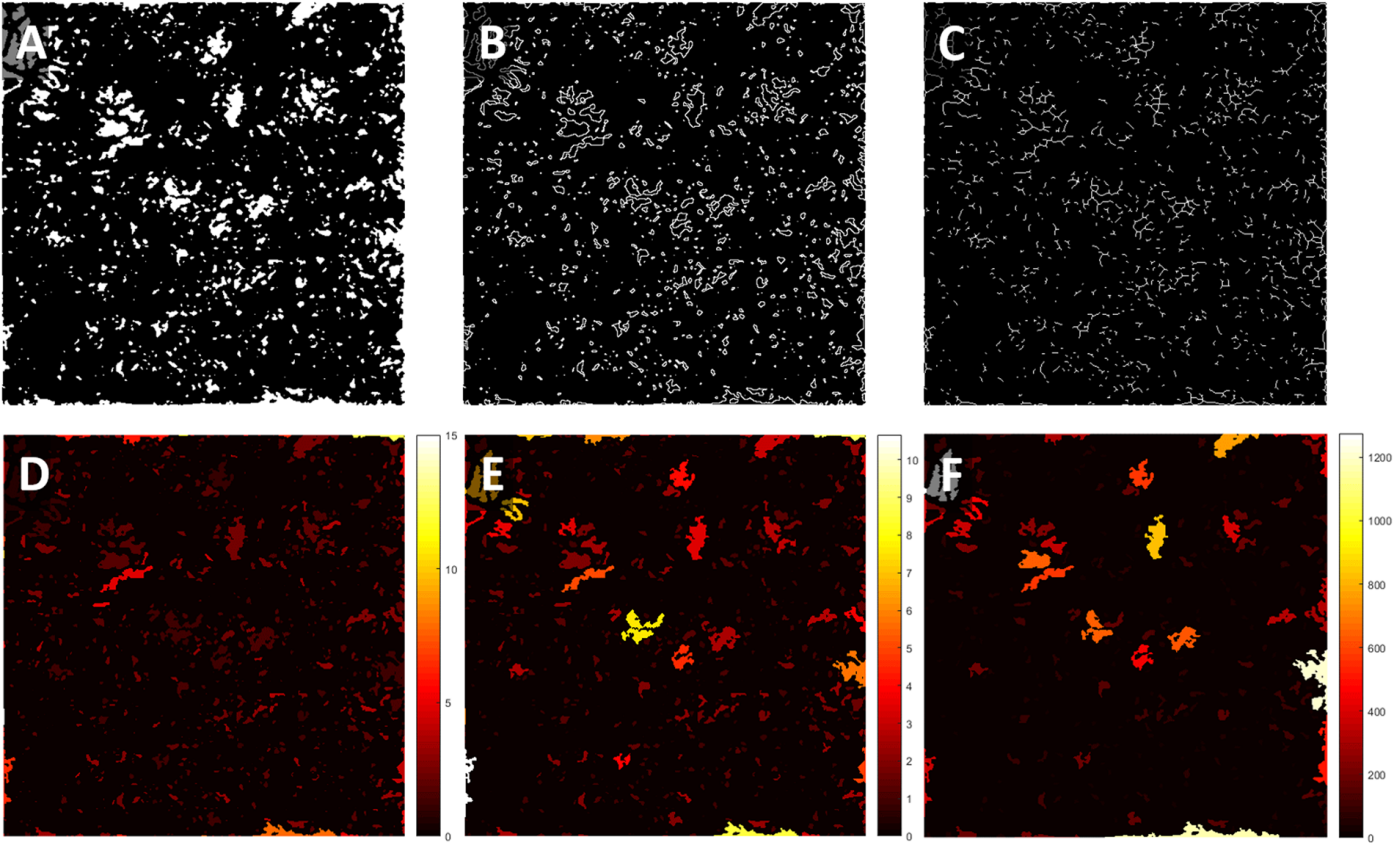
Visual illustration of quantification maps derived from CC FD map. (**A**) Binary CC FD map, white pixels represent identified FDs; (**B**) CC FD perimeter map, white pixels represents identified perimeter of FDs; (**C**) CC FD length map, white pixels represents identified center length of FDs; (**D**) CC FD aspect ratio map, color bar represents calculated aspect ratio of FDs, unit-less; (**E**) CC FD complexity map, color represents calculated complexity index of FDs, unit-less; (**F**) CC FD size map, color bar represents calculated size of FDs, unit is µm^2^.

We designed a series of quantitative indices specifically for CC with an emphasis on FD. Similar to previously reported study^43^, we have generated binary CC FD map, CC FD perimeter map and CC FD skeleton map as shown in Fig. 3A–C. Additionally, we have calculated the size, aspect ratio and complexity index of individual CC FDs and color coded the values of each CC FD onto the binary CC FD map (Fig. 3D–F). Correspondingly, we defined a series of quantitative indices calculated from these six maps.

Flow deficit density (FDD) is defined as a unit-less ratio of the image area occupied by CC FDs to the total image area in the CC FD binary map:

2 $$FDD=\frac{{\sum }_{i=1,\,j=1}^{n}\,\,{A}_{(i,j)}}{{\sum }_{i=1,\,j=1}^{n}\,\,{X}_{(i,j)}}$$

where *A*_(*i*,*j*)_ represents white pixels in the binary CC FD map (Fig. 3A) and *X*_(*i*,*j*)_ represents all pixels in the binary CC FD map. (i, j) Are the pixel coordinates in the OCTA image (assuming a n × n pixel array). A higher FDD value corresponds to more severe CC vasculature loss. We believe this parameter would be useful in most diseases.

Flow deficit aspect ratio index (FDARI) is defined as the average of all individual FD aspect ratios of major axis length to minor axis length.

3 $$FDARI=\frac{{\sum }_{i=1}^{x}\,\frac{{M}_{(i)}}{{N}_{(i)}}}{x}$$

Here, *M*_(*i*)_ represents the estimated major axis length of each individual FD and N_(*i*)_ represents the estimated minor axis length. *i* represents all individual FDs and x is the total number of FDs. FDARI can be used to represent the geometric shape of FDs and therefore contains morphological information. FDARI is similar to the axis ratio in quantifying foveal avascular zone (FAZ)^44^, and this index represents the irregularity and acircularity of CC FDs.

Flow deficit complexity index (FDCI) describes the morphological complexity of detected FDs, defined as:

4 $$FDCI=\frac{{\sum }_{i=1}^{x}\,\frac{{{P}_{(i)}}^{2}}{4\pi {A}_{(i)}}}{x}$$

where *P*_(*i*)_ represents the perimeter of each individual FD and *A*_(*i*)_ represents the area of each individual FD. *i* Represents all individual FDs and x is the total number of FDs. FDCI describes the morphological complexity of FDs with an emphasis on the boundaries. A higher FDCI value corresponds to a more complicated shape of CC FDs.

Flow deficit number (FDN) is defined as the total number of FDs detected and Flow deficit size (FDS) is defined as the averaged FD size of all FDs detected:

5 $$FDS=\frac{{\sum }_{i=1,\,j=1}^{n}\,\,{A}_{(i,j)}}{FDN}$$

where *A*_(*i*,*j*)_ represents white pixels in the binary CC FD map (Fig. 3A). (i, j) are the pixel coordinates in the OCTA image (assuming n × n pixel array). An increase in FDN and FDS may indicate increasing number and expansion of non-perfusion areas or FDs. FDN and FDS together can resolve the differences between either a small number of large FDs indicating centralized and localized CC loss, or a large number of small FDs indicating scattered global CC loss, and hence provide more detail than FDD alone.

### Statistical analysis

Statistical analyses were performed using MATLAB (R2016b; MathWorks, Inc, Natick, Massachusetts, USA). Results were expressed using mean and 95% confidence interval (CI). Paired t-test were used to compare the FD measurements from different number of averages and two sample t-test was used to compare the FD measurements of macular CC and equatorial CC. Pearson correlation was used for correlation tests and Bland-Altman analysis was used for agreement tests.

## Results

Eleven eyes from six normal subjects (age: 26–67, 4 males, 2 females) were recruited in this study. Each subject was scanned at several locations from the fovea to the equatorial region and with five repeated volume scans.

### Improving visualization of CC with registration and averaging algorithm

**Figure 4:**
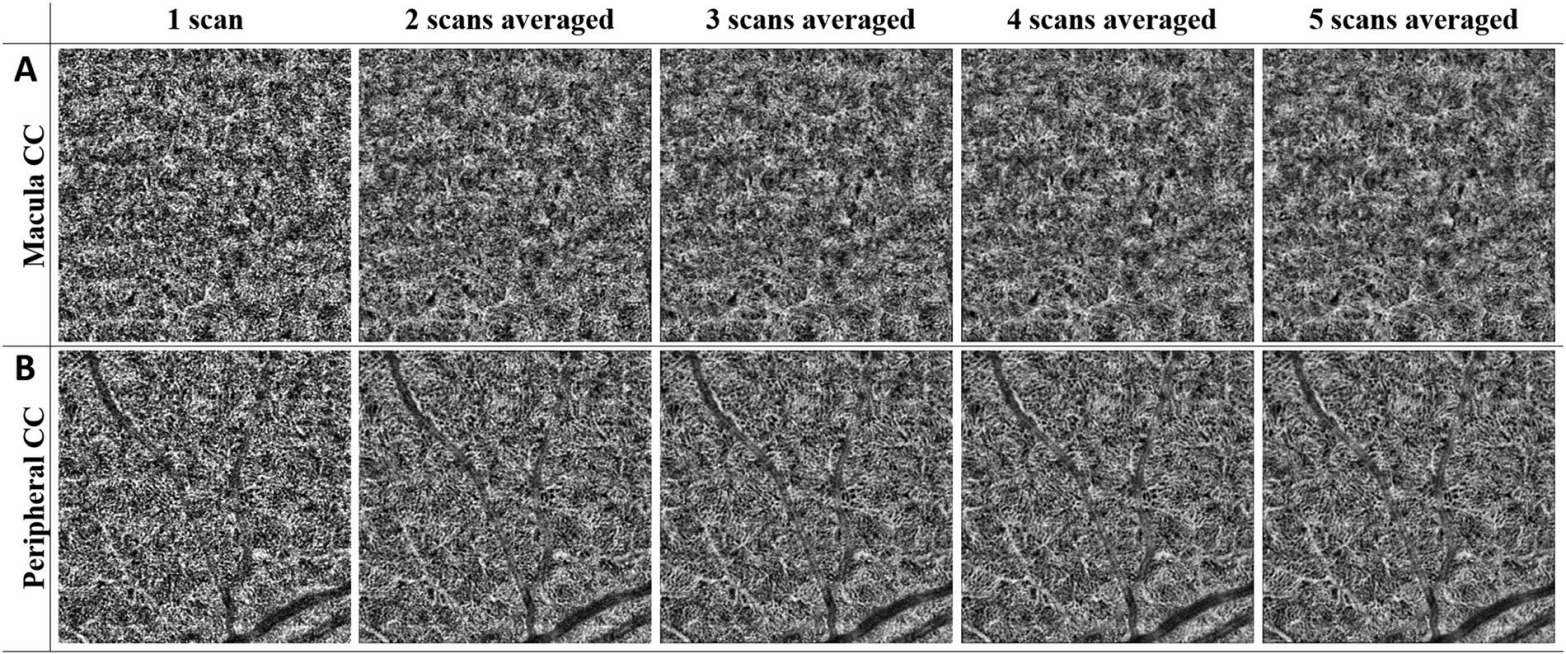
Improved visualization of macular CC and equatorial CC can be achieved by registration and averaging algorithm. Top row (**A**) macular CC of single scan, 2 scans averaged, 3 scans averaged, 4 scans averaged, and 5 scans averaged, respectively. Bottom row (**B**) equatorial CC of single scan, 2 scans averaged, 3 scans averaged, 4 scans averaged and 5 scans averaged, respectively.

**Figure 5:**
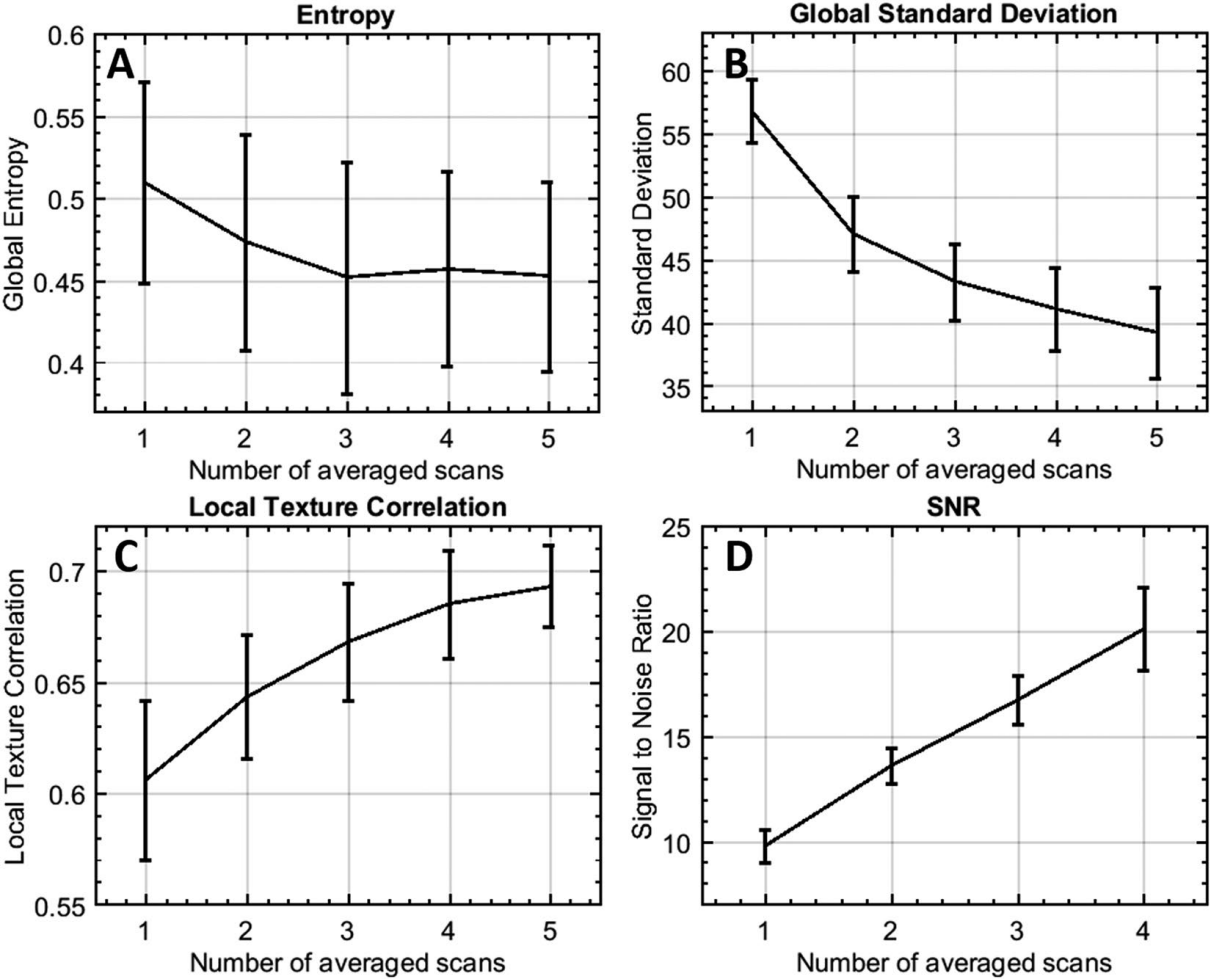
Performance evaluation of registration and averaging algorithm against the number of averaged scans. (**A**) Global entropy (0–1); (**B**) global standard deviation (y-axis ranging from 0–255); (**C**) local texture correlation (y-axis ranging from 0–1); (**D**) PSNR using five averages as reference.

Representative averaging effects on OCTA images of macular and equatorial CC were shown in Fig. 4. Increasing averaging numbers largely improved the continuity of blood vessels and the overall contrast of the whole image. Furthermore, parameters of global entropy, global standard deviation, local texture correlation and PSNR confirmed the improvement from registration and averaging algorithm (Fig. 5). With increasing number of repeats, the global standard deviation decreases as CC images become smoother. Similarly, local texture correlation and PSNR also increase with the increase of repeated scans. On the other hand, global entropy, a statistical measurement of the randomness, decreases with the increase of repeated scans.

**Figure 6:**
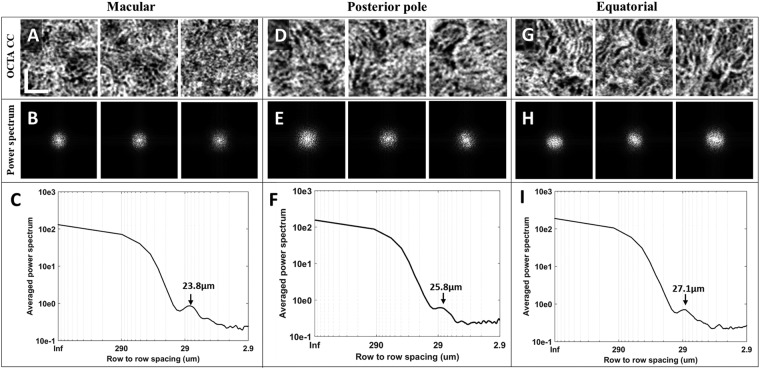
Power spectrum analysis of the inter-capillary distance for macular CC, posterior pole CC and equatorial CC, respectively. (**A**) Three selected 650 µm *650 µm regions of macular CC; (**B**) 2D power spectrum of macular CC in A; (**C**) example radially averaged power spectrum plot of macular CC. (**D**) Three selected 650 µm ∗ 650 µm regions of posterior pole CC (~4 mm away from fovea); (**E**) 2D power spectrum of macular CC in D; (**F**) example radially averaged power spectrum plot of posterior pole CC. (**G**) Three selected 650 µm ∗ 650 µm regions of equatorial CC; (**H**) 2D power spectrum of equatorial CC in G; (**I**) example radially averaged power spectrum plot of equatorial CC. Scale bar represent 200 µm.

**Figure 7:**
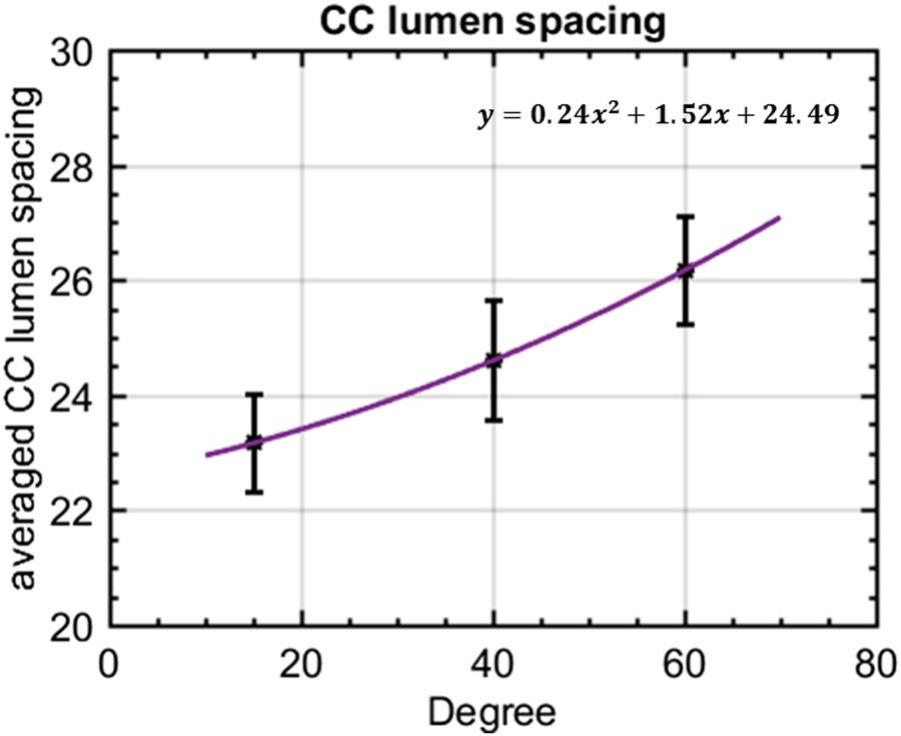
The inter-capillary distance increases as the increase of radial distance with respect to central fovea (depicted as degrees). X-axis indicates the estimated relative locations.

Intercapillary distance is a key parameter to evaluate the normality of CC. An improved visualization of CC will result in more accurate measurement of ICD. OCTA images of three selected scans in macular, posterior pole and equatorial segments showed the distinguishing morphologies of CC (Fig. 6). This observation was consistent with previous histological anatomies^5,6^. Averaged ICD of CC was 22.2 µm, 23.5 µm and 23.8 µm for the three scans in macular, 23.2 µm, 23.8 µm and 25.8 µm in posterior pole, and 25.1 µm, 26.2 µm and 27.1 µm in equatorial regions. Corresponding 95% confidence intervals were reported in Table 1. Polynomial curve fitting was then applied for ICD along macular, posterior pole and equatorial locations. The ICD is around 24 µm in macular regions and become larger toward equatorial regions as indicated by power spectrum analysis (Fig. 7).

**Table 1:** Descriptive statistics of CC lumens spacing using spatial power spectrum analysis.

	Mean (95% CI)		
	Macular CC	Posterior pole CC	Equatorial CC
Average CC lumens spacing (µm)	23.17 (21.05–25.28)	24.60 (21.97–27.22)	26.14 (23.64–28.62)

### Quantitative indices with multiple averaging

**Figure 8:**
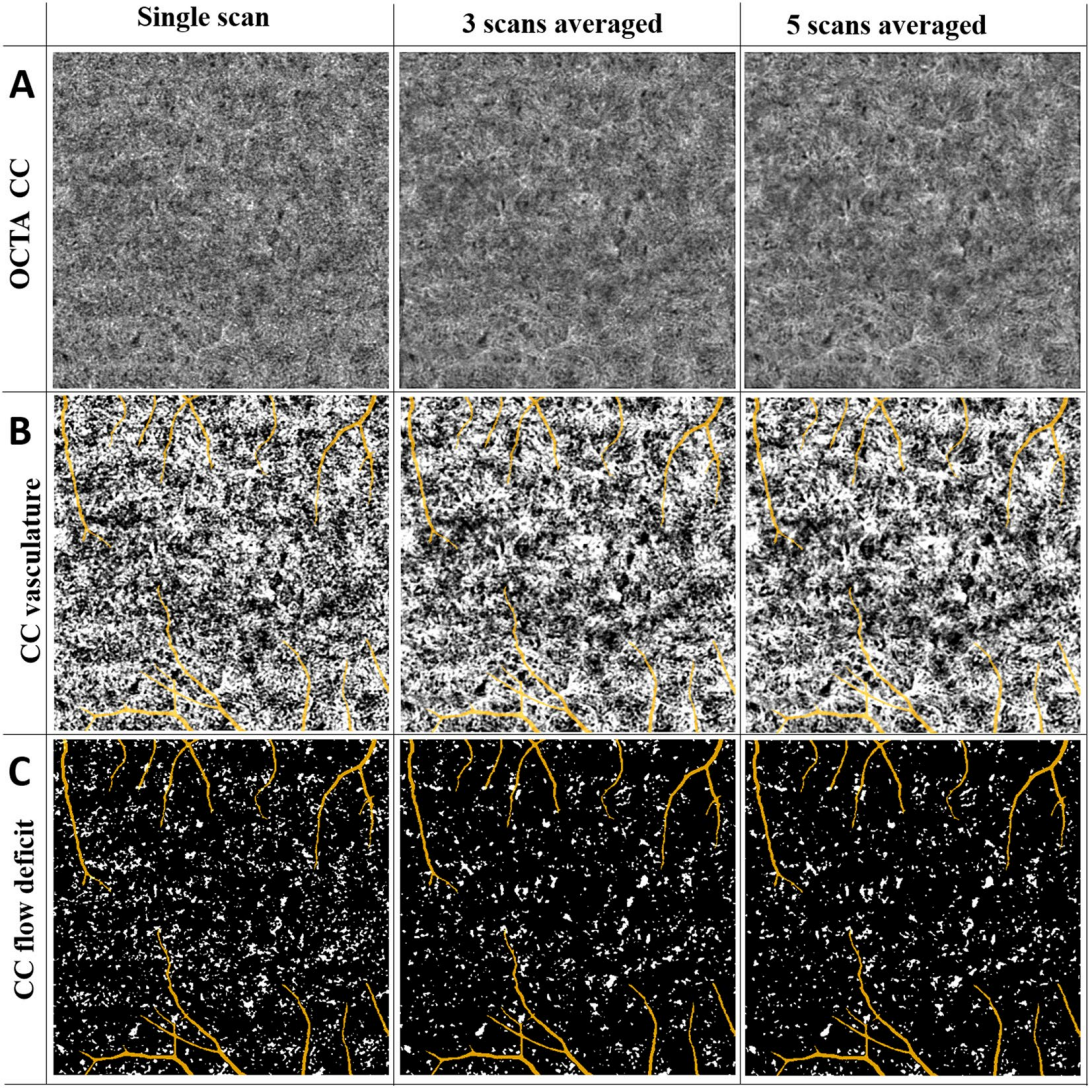
Visual illustration of CC FDs detection with averaging for the scans obtained from macular region. (Panel A) Macular CC OCTA images with single scan, three scans averaged, and five scans averaged, respectively. (Panel B) Macular CC vasculature segmented by fuzzy c-means algorithm with single scan, three scans averaged, and five scans averaged, respectively. (Panel C) Segmented macular CC FD with single scan, three scans averaged and five scans averaged, respectively.

**Figure 9:**
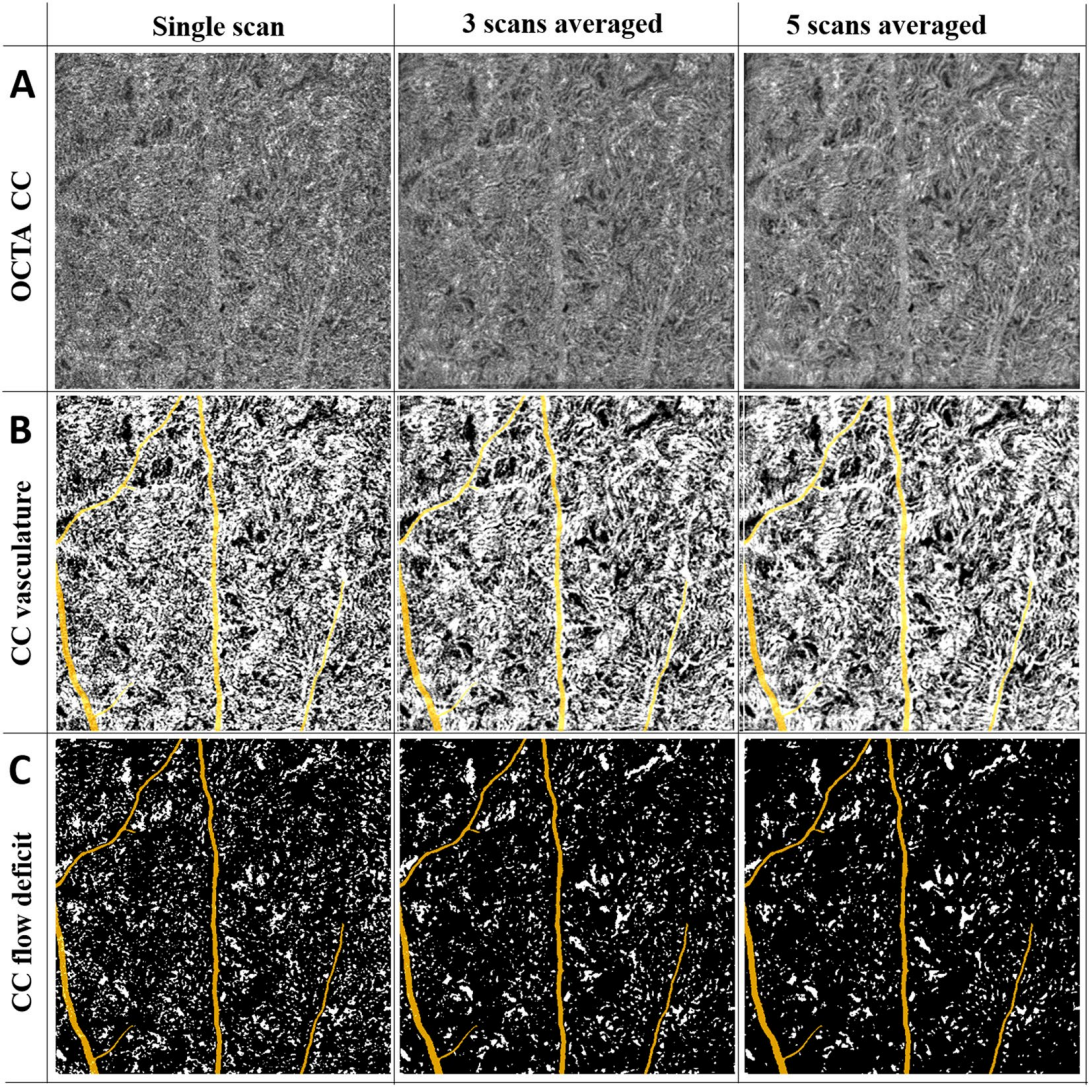
Visual illustration of CC FDs detection with averaging for the scans obtained from equatorial region. (Panel A) Macular CC OCTA images with single scan, three scans averaged, and five scans averaged, respectively. (Panel B) Macular CC vasculature segmented by fuzzy c-means algorithm with single scan, three scans averaged, and five scans averaged, respectively. (Panel C) Segmented macular CC FD with single scan, three scans averaged, and five scans averaged, respectively.

We further evaluated the quantitative indices with multiple averaging on macular CC and equatorial CC. Figures 8 and 9 show a series of representative averaged CC images, CC vasculature and detected FDs in macular and equatorial CC, respectively. Projection artifacts were removed as colored in yellow. Averaging multiple scans helped smoothing CC vasculature and increased the continuity of blood vessels. For FD detection, averaged scans reduced speckle noises which resulted in less FDs.

**Figure 10:**
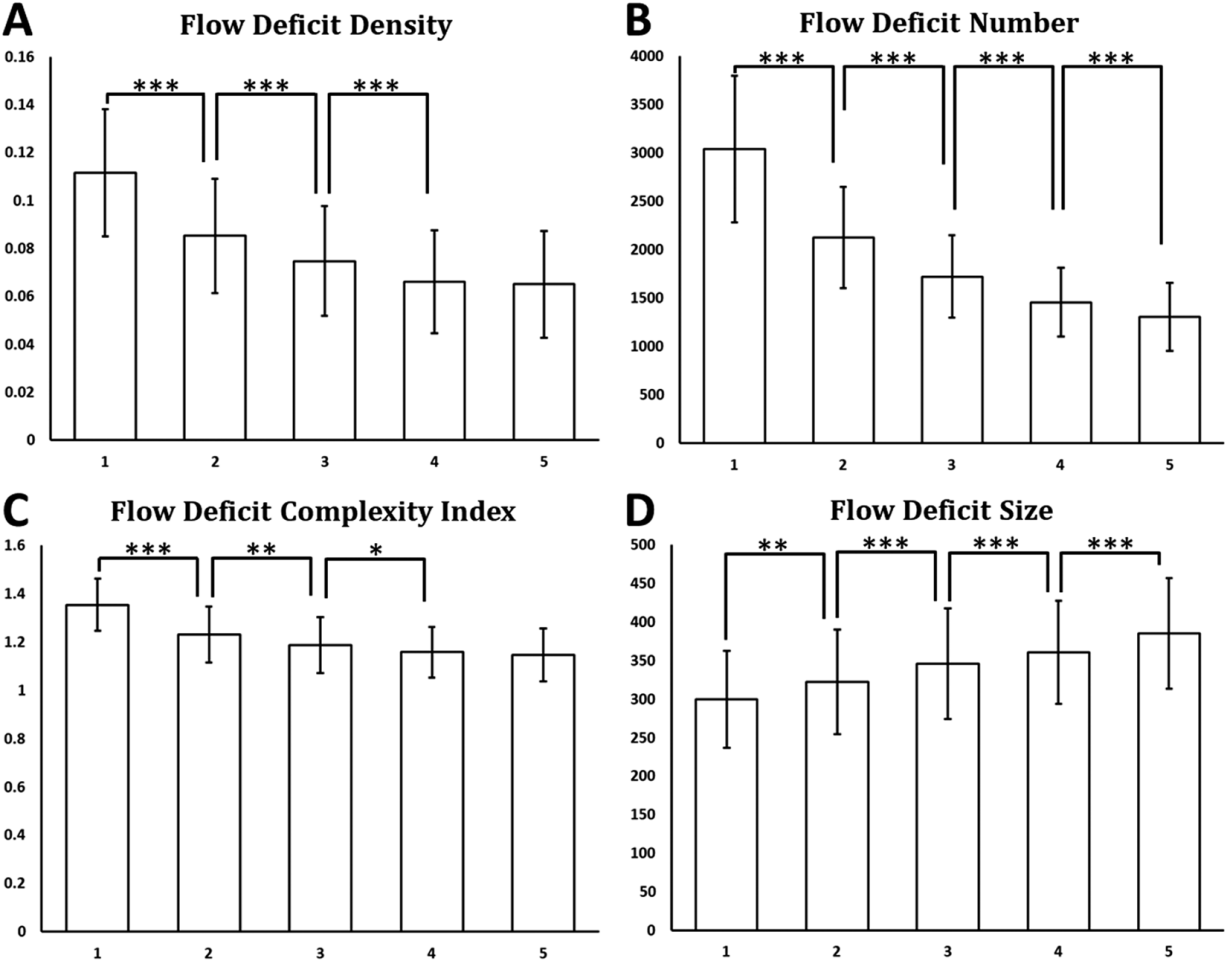
Quantitative analysis of CC FDs with multiply averaged OCTA images. X-axis represents number of images averaged. Y-axis in (**A**) Flow deficits density; (**B**) Flow deficits number; (**C**) Flow deficits complexity index; and (**D**) Flow deficits size. ^*^Denotes p < 0.05, ^**^Denotes p < 0.01 and ^***^Denotes p < 0.001.

FDD, FDN, FDCI, FDN and FDARI were calculated using single-scan OCTA images of CC as well as averaged OCTA images with averaging number of up to five (Fig. 10). Significant paired t test results were denoted with *(p < 0.05), **(p < 0.01) and ***(p < 0.001) on corresponding plots. For FDD and FDCI, we found that each time of adding one more scan to register and average decreased significantly until four averaged scans were reached. Five-scan averaged CC OCTA images did not yield to significant difference in FDD or FDCI compared to four-scan averaged ones. FDN and FDS showed significant difference every time we increased the number of repeated scans for averaging. We did not find any significant differences in FDARI with the increased number of the repeated scans for averaging (data not shown).

**Figure 11:**
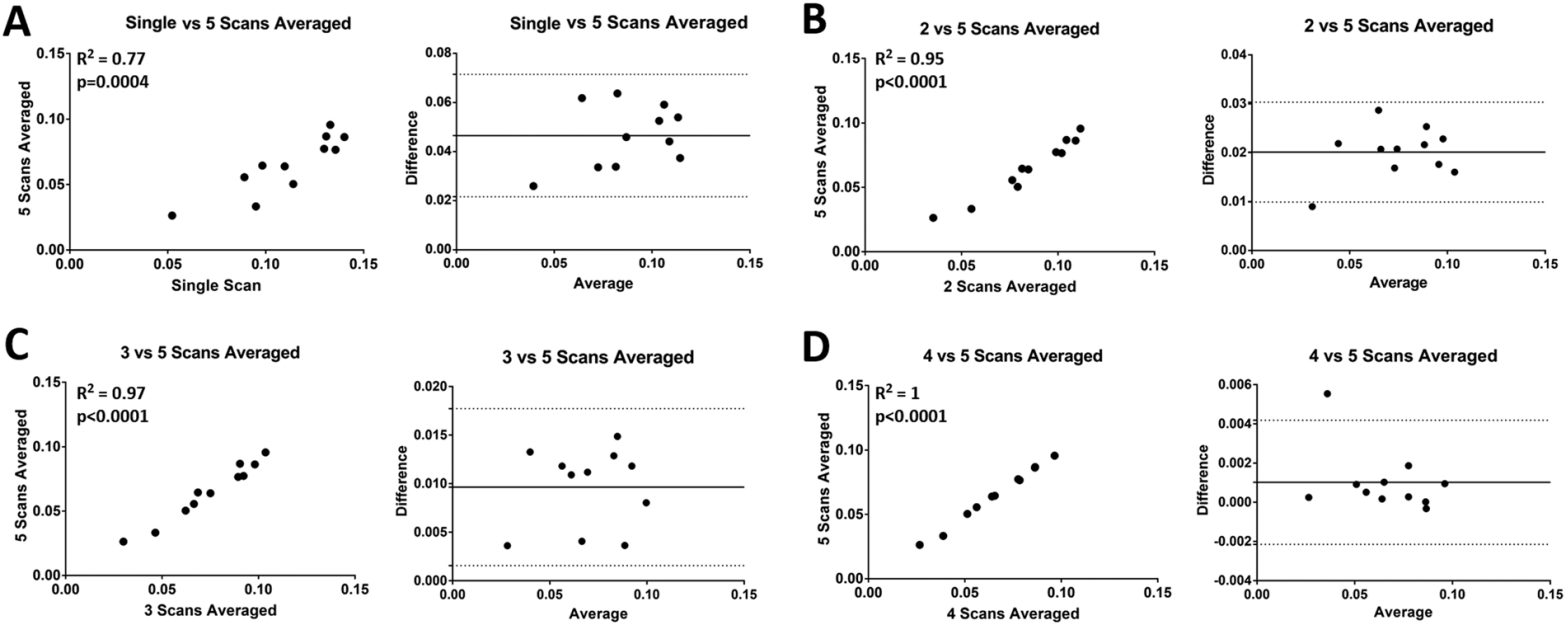
Correlation and Bland-Altman plots of CC FDD with multiple averaged scans. (**A**) Comparing single scan CC FDD results with 5 scans averaged CC FDD results; (**B**) comparing 2 scans averaged CC FDD results with 5 scans averaged CC FDD results; (**C**) comparing 3 scans averaged CC FDD results with 5 scans averaged CC FDD results; (**D**) comparing 4 scans averaged CC FDD results with 5 scans averaged CC FDD results.

For FDD, which is currently widely used in the literature for CC evaluation, correlation and Bland-Altman agreement plots were produced with increasing numbers of repeated scans for averaging (Fig. 11). Five-scan averaged CC were used as a reference for comparison. Correlation was good between single scan and five-scan averages (*R*^2^ = 0.77), with a mean difference of ~0.046 and limit of agreement of 0.021–0.070. Registration and averaging largely improved the correlation, with *R*^2^ increased to 0.95, 0.97 and 1.00 for two, three and four averages, respectively.

### Quantitative assessment of macular CC and equatorial CC

CC under macular and CC in the equatorial regions have been reported to have different vasculature patterns^8^ and this was also confirmed in visual presentations and measured ICDs discussed above. Quantitative assessment of CC provides more details of the differences in the two locations (Table 2). For FDD, we found that macular CC has a significantly lower FDD compared to equatorial CC (5.35% vs 8.45%, p = 0.002). Equatorial CC were found to have larger FDS than macular CC (432.20 *um*^2^ vs 358.96 *um*^2^, p < 0.0001). No significant difference was found in FDN between equatorial CC and macular CC. Moreover, for the morphology comparisons, equatorial CC showed a higher FDARI than macular CC (2.16 vs 1.95, p = 0.01). This indicates that the FDs are more elongated in equatorial regions which agrees with histological observations^5–7^. FDCI also yielded to a higher value in equatorial CC than macular CC (1.22 vs 1.11, p = 0.02).

**Table 2:** Descriptive stats of quantitative comparison of CC FDs in macular and equatorial regions.

Quantitative indices	Mean (95% CI)	
	Macular CC	Equatorial CC
FDD (%)^*^	5.35 (3.76–6.94)	8.45 (7.48–9.42)
FDS (µm^2^)^*^	358.96 (277.45–440.46)	432.20 (371.48–492.91)
FDN	1191 (760–1622)	1538 (1374–1704)
FDARI^*^	1.95 (1.90–1.99)	2.16 (1.97–2.33)
FDCI^*^	1.11 (1.03–1.19)	1.22 (1.15–1.29)

^*^Denotes p < 0.05.

## Discussion

In this study, we introduced and validated a novel approach of registration and averaging to improve the visualization of CC, and provided multiple indices for quantitative assessment of CC using SS-OCTA. Our results showed that more averages produce better quality CC images and more distinct features of CC vasculature, and are in accordance with previous histology reports^5–7^. ICD was measured to be around 24 µm under macula and increasing toward equatorial regions. Our five metrics for CC quantification were validated with multiple averaging and showed significant differences of CC under macula and in equatorial regions.

Unlike prior studies^27^ that relied on retinal vasculature for registration and averaging, we performed registration directly on CC vasculature. Since intact retinal vasculature is not required to perform averaging, our method can be applied in various diseased subjects such as DR subjects with severe vasculature loss, subjects with artery or vein occlusions and so on. This is illustrated with the BSCR patient (Fig. 2). Moreover, we can register and average CC under the FAZ, which could be of great importance to study AMD, whereas registration based on retinal vasculature cannot achieve this due to the avascular property of the FAZ.

Limited lateral resolution and sampling rate as well as speckle noises are the major challenges of commercial OCTA systems in imaging CC *in vivo*. In this study, we were able to improve the visualization of CC by registering and averaging multiple OCTA scans. Even though this does not improve the system’s lateral resolution, it is very successful in reducing speckle noises that are prevalent in single scan images. We have demonstrated that registering and averaging multiple scans is a practical tool to improve CC imaging on commercial OCTA systems. According to the correlation and agreement analysis, multiple-scan averaged CC images are more ideal than single scan CC images. However, single-scan CC images could still be meaningful in clinical studies where multiple averages are difficult to collect, since CC quantification based on single-scan image correlates well with CC quantification based on 5-scan averaged images (*R*^2^ = 0.77), yet with a mean bias of ~4.5%.

In this study, we have also designed a series of quantitative indices to describe CC FDs: FDD, FDN, FDS, FDARI and FDCI. FDD is the density of FDs that has been applied in previous studies of CC quantification. FDN is the number of total FDs detected while FDS is the averaged size of FDs detected. When two cases have the same FDD, FDS and FDN together can tell the differences between centralized and localized CC loss from scattered global CC loss. FDARI describes the aspect ratio of CC FDs, and similar concepts have been successfully used to detect abnormal FAZ in retina^44^. FDCI described the morphological complexity of FDs, with an emphasis on the boundaries. FDARI and FDCI together could indicate the shape and circularity of FDs. These indices could be useful to study the loss of CC in diseased cases as well as to longitudinally monitor the changes of CC in treatment monitoring and patient follow up. Encouragingly, our CC quantitative indices showed significant differences between macular CC and equatorial CC. This demonstrates that our quantification can detect the subtle difference in different CC vascular networks, indicating that it is highly possible that our quantitative analysis would be capable of detecting CC abnormalities in diseased cases too.

There are also limitations in our study. Firstly, our sample size is small (6 subjects). To rigorously validate our method a larger sample size will be needed for implementation in clinical settings. Secondly, we have not applied our analysis on diseased subjects clinically on a large scale. There could be potential downsides of averaging. For example, averaging might reduce low speed and intermittent CC blood flow that has a similar pattern as speckle noises. Generally speaking, averaging multiple CC scans would increase the specificity of detecting CC vasculature and decrease the sensitivity. It is unlikely such potential downside would play a significant role in quantitative analysis but future studies on different pathological cases are warranted for further proof.

## Conclusion

We have shown that the three-step registering, and averaging algorithm based on multiple OCTA *en face* images of CC can largely improve the visualization of CC vasculature. The designed five indices of quantitative assessment of CC as well as the corresponding functional maps demonstrated their capacity in detecting subtle differences in CC vascular networks at different locations. Overall, this technique hold promises for future quantitative assessment of CC in normal and diseased human eyes *in vivo*.
